# Increased Levels of Anti-*Anisakis* Antibodies During Hospital Admission in Septic Patients

**DOI:** 10.3390/antib13040096

**Published:** 2024-11-27

**Authors:** Juan Carlos Andreu-Ballester, Amparo Navarro, Miguel Angel Arribas, Moises Rico, Laura Albert, Carlos García-Ballesteros, Lorena Galindo-Regal, Rosa Sorando-Serra, Francisca López-Chuliá, Federico Peydro, Marta Rodero, Juan González-Fernández, Carmen Cuéllar

**Affiliations:** 1 FISABIO Foundation–Public Health of Valencia, 46015 Valencia, Spain; jcandreuballester@outlook.com (J.C.A.-B.); lopez_frachu@gva.es (F.L.-C.); 2 Parasitic Immunobiology and Immunomodulation Research Group (INMUNOPAR), Complutense University, 28040 Madrid, Spain; mrodero@ucm.es (M.R.); juangonzalez@ucm.es (J.G.-F.); 3 Critical Care Department, Arnau de Vilanova University Hospital, 46015 Valencia, Spain; amparonavarroeduardo@gmail.com (A.N.); marribas42@gmail.com (M.A.A.); moises@comv.es (M.R.); lauraalbertrodrigo@gmail.com (L.A.); federo42@hotmail.com (F.P.); 4 Laboratory of Molecular Biology, Arnau de Vilanova University Hospital, 46015 Valencia, Spain; garcia_carbar@gva.es (C.G.-B.); loregal@msn.com (L.G.-R.); 5 Emergency Department, Arnau de Vilanova University Hospital, 46015 Valencia, Spain; rosasorando@gmail.com; 6 Hematology Department, Arnau de Vilanova University Hospital, 46015 Valencia, Spain; 7 Microbiology and Parasitology Department, Complutense University, 46015 Madrid, Spain

**Keywords:** anti-*Anisakis* antibodies, sepsis, αβ T cells, γδ T cells, apoptosis, ELISA, mortality

## Abstract

Background/Objectives: In a previous study, we described elevated anti-*Anisakis* IgG levels in septic patients in relation to disease severity. In this study, our objective was to analyze the evolution of anti-*Anisakis* immunoglobulins in septic patients during hospital admission and their association with αβ and γδ T cell subsets. Methods: We recruited 80 subjects: 40 patients with sepsis and 40 controls. αβ and γδ T cells were analyzed using flow cytometry. Apoptosis was also assessed, and anti-*Anisakis* antibodies were measured by ELISA in the sera of patients with sepsis and controls. Results: In the second analysis (7–10 after sepsis evolution), an increase in all specific antibody isotypes was identified in individuals with septic shock, except IgE. The levels of anti-*Anisakis* IgG and IgA were higher in the subjects with sepsis in the first analysis and continued to increase in the second analysis compared with the healthy control subjects. There was an increase in anti-*Anisakis* IgG and IgA levels in surviving patients and an increase in IgA levels in non-surviving patients. A rise in specific IgG and IgE levels was noted in the second analysis of patients with sepsis with αβ CD3+ T cell deficiency. Patients without γδ T cell deficiency had increased anti-*Anisakis* IgA levels 7–10 days after admission. Conclusions: Our results suggest a previous infection by *Anisakis* that could be involved in the subsequent septic process and be related to patients who have negative cultures in which the pathogen causing sepsis has not been identified.

## 1. Introduction

Sepsis continues to be a serious public health problem, with an estimated incidence of approximately 700 cases/100,000 people/year worldwide, in both developed and developing countries [1,2]. It is known that in the evolution of sepsis, after the first process of cytokine storm, a second state of immunoparalysis occurs, in which the body’s immune cells decrease significantly. This phenomenon is attributed to the apoptosis of immune cells and correlates with the severity and mortality of sepsis, especially in relation to γδ T cells [3,4,5,6,7]. This phase of immunosuppression is often linked to a substantial rise in positive blood cultures, particularly for opportunistic bacteria, fungi, and parasites. Furthermore, the majority of mortality occurs five days or more after sepsis onset [8]. We previously detected higher levels of IgE anti-*Encephalitozoon cuniculi*, an opportunistic fungus belonging to microsporidia, in the serum of patients with sepsis. Anti-*E. cuniculi* IgE in septic patients was associated with a reduction in αβ and γδ T cells in peripheral blood. This decline was more pronounced in the CD3+CD56+ γδ T cell subset [9].

Similarly, reactivation of latent viruses has been demonstrated in patients with prolonged sepsis, as observed in transplant patients receiving immunosuppressive treatments. This is consistent with the immunoparalysis phase of sepsis.

Human cytomegalovirus (HCMV or HHV-5), Epstein–Barr virus (EBV or HHV-4), herpes simplex virus (HSV-1 or HHV-1 and HSV-2 or HHV-2), human herpesvirus 6 (HHV-6), and polyomavirus have higher rates in patients with sepsis than in healthy controls. Reactivation has been documented in the late stages of sepsis, beyond the 6th day, and was found to be associated with a notable increase in the rate of morbidity and mortality [10,11,12].

*Anisakis*, a genus of parasitic nematodes, infects more than one billion people worldwide through the intake of inadequately heated/frozen seafood. Live larvae generally penetrate gastrointestinal mucosa, causing acute pain or allergic manifestations, among other symptoms. Furthermore, the infections with parasitic helminths are associated with non-tolerance of oral antigens, increased vulnerability to secondary infections, and reduced vaccine efficacy [13,14]. However, humans are not definitive hosts for this parasite, and *Anisakis* parasitism is accidental and typically acute or “intermittent” (repeated acute parasitism by *Anisakis*). As a result, *Anisakis* may not possess the immunoregulatory features commonly observed in other parasitic worms [15].

The immunological response mechanisms against this parasite have been studied, including the formation of granulomas, presenting both Th1 and Th2 reactions, although with a great preponderance of the latter, and curiously with hardly any expression of IL-2 and INF-γ [16]. The production of antibodies against the parasite antigens, crude extract, and excretory–secretory products follows a typical evolution with a maximum increase in IgM on the first day, progressively decreasing thereafter, while the levels of IgA, IgG, and IgE increase one month after infection and still remain high at six months [17].

Antibodies against *Anisakis* are produced first by the stimulation of excretion–secretion products produced by live larvae, and then against somatic antigens released after the death and rupture of the larvae. It is important to keep in mind that larval death begins to occur about week after infection [16,18]. In any case, it is unknown to what extent some of its components can remain in the body and cause immunomodulatory effects in transient immunodeficiency states, as occurs in sepsis. In a previous study, we described high anti-*Anisakis* IgG levels in patients with sepsis, with an increase in subjects with positivity in relation to severity, reaching 30% in patients with septic shock [19].

Our objective was to analyze the evolution of anti-*Anisakis* antibodies in patients with sepsis during hospital admission and their relationship with the cellular immunity of αβ and γδ T cell subsets.

## 2. Materials and Methods

### 2.1. Study Type and Population

In this prospective study, we enrolled a total of 80 participants: 40 septic patients and 40 healthy individuals serving as the control group. The cases and controls were matched based on sex and age, with a variation of ±5 years. The diagnosis of all patients was made according to the most recent criteria for defining and managing sepsis [20]. The Emergency and Intensive Care departments of Lliria Hospital and Arnau de Vilanova University Hospital (Valencia, Spain) provided the samples from septic patients over a one-year period starting in March 2019. To qualify for the study, patients were required to fulfill the following inclusion criteria: they could have neither immune system deficiencies nor autoimmune or inflammatory diseases; must not have been immunized with a vaccine in the last three months, and should not be undergoing immunosuppressive therapy. Healthy control subjects were required to share similar characteristics with the patients, in addition to not having any acute infectious diseases. This study was approved by the Institutional Review Board of Arnau de Vilanova University Hospital (Valencia, Spain).

### 2.2. Methods of Blood Sample Analysis

A cell counter (LH750 Beckman Coulter, Inc., Fullerton, CA, USA) was used for the blood cell counts after patients were admitted to the Emergency and Intensive Care units. Samples were collected upon patient admission and between days 7 to 10 following the progression to sepsis.

#### 2.2.1. Evaluation of γδ and αβ T Cells and Apoptosis

Mononuclear cells (MNCs) were extracted and concentrated from EDTA–anticoagulated blood by centrifugation on a density gradient. Lymphoprep™ (Palex Medical SA, Barcelona, Spain) was utilized, spinning at 3500 rpm for 20 min. After two washing steps with phosphate-buffered saline (PBS), the cells were re-suspended in 200 μL of binding buffer contained in the ANNEXIN V-FITC/7-AAD Kit (Beckman Coulter, Inc., Miami, FL, USA) in the presence of calcium ions (Ca^2+^).

The monoclonal antibodies employed were the following: anti-TCR PANαβ-PE (clone: IP26A), anti-TCR PANγδ-PE (clone: IMMU 510), CD56-PC7 (clone: N901 (NKH-1)), CD4-APC A750 (clone: 13B8.2), CD3-APC A700 (clone: UCHT1), CD8-PB (clone: B9.11), and CD45-KRO (clone: J33), all manufactured by Beckman Coulter, Inc., Miami, FL, USA.

After a 10 min incubation, a “Fix-and-Lyse” solution (Versalyse plus IOT3, from Beckman Coulter, Inc., Miami, FL, USA) was prepared and 1 mL of this mixture was added to the MNC pellet, followed by a 20 min incubation at room temperature in the dark. Subsequently, 2 mL of PBS was added, and the mixture was centrifuged for 5 min at 1500 rpm. The supernatant was carefully aspirated, and the cell pellet was re-suspended in 0.5 mL of PBS.

A total of 100,000 events were collected using a multiparameter Navios flow cytometer (Beckman Coulter, Inc., Miami, FL, USA) and subsequently analyzed with Kaluza software 2.3.

#### 2.2.2. Extraction of Anisakis Antigen and Specific Antibody Detection

*Anisakis* L3 larvae were obtained from blue whiting (*Micromesistius poutassou*) and were homogenized through sonication and extraction in PBS, as previously outlined [21].

ELISA plates (Costar, Corning, NY, USA) were coated with 10 µg/mL of total larval antigen. Human serum samples were diluted to 1/100 in PBS–Tween containing 0.1% BSA and then incubated. For detection, peroxidase (HRP)-conjugated goat anti-human immunoglobulins (Ig’s, IgM, IgG, or IgA; Biosource International, Camarillo, CA, USA) were utilized [17,22]. For the determination of IgE, serum samples were diluted to 1/2. Following this, a murine anti-human IgE monoclonal antibody (IgG1Ƙ, E21A11, INGENASA, Madrid, Spain) was introduced, along with a goat anti-murine IgG1 antibody conjugated with HRP (Life Technologies, Grand Island, NY, USA). The positivity threshold for anti-*Anisakis* antibody levels was defined as the optical density exceeding the mean plus one standard deviation of values obtained from healthy individuals.

### 2.3. Statistical Analysis

The Mann–Whitney U test, a non-parametric method, was employed to compare the counts of T cell subsets. Differences in mean values between the first analysis (at admission) and the second analysis (after 7–10 days) were evaluated using the Wilcoxon non-parametric test. To compare the differences between surviving and non-surviving septic patients, the Mann–Whitney U test was again applied. The McNemar test was used to analyze changes in anti-*Anisakis* antibody positivity from the first (at admission) to the second (after 7–10 days) analysis. Correlation studies were conducted using Pearson’s test to compare the numbers of T cell subsets with specific antibodies against *Anisakis*. The deficiency of αβ and γδ T cell subsets was calculated based on a previously established method [19]. A significance level of *p* < 0.05 was set. Data analysis was performed using the IBM SPSS Statistics 19.0 (SPSS Inc., Chicago, IL, USA), and figures were generated with GraphPad Prism 8.0.0 for Windows (GraphPad Software, San Diego, CA, USA).

## 3. Results

The characteristics of the patients with sepsis are summarized in Table 1.

### 3.1. Specific Antibodies Against Anisakis

**Figure 1:**
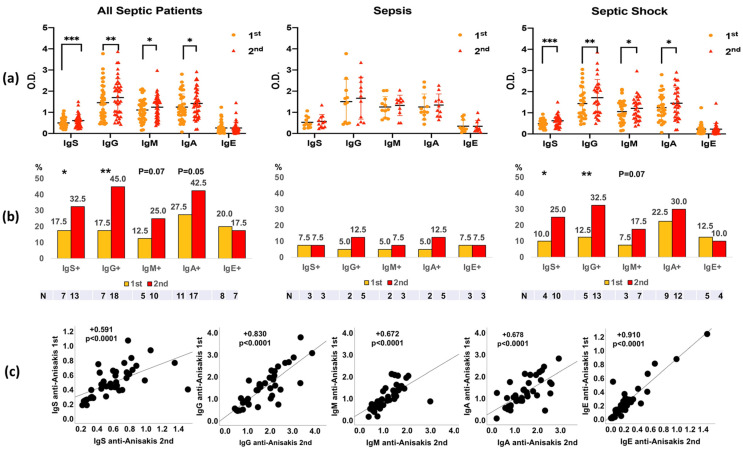
Differences in anti-*Anisakis* immunoglobulins between 1st (at admission) and 2nd analysis (after 7–10 days) in all septic patients (n = 40), sepsis patients (n = 11), and septic shock patients (n = 29) (Panel (**a**)). Values are expressed as mean Optical Density (O.D.). T bars denote the standard deviation. The Wilcoxon test was used. (*** *p* < 0.001, ** *p* < 0.01 and * *p* < 0.05). Percentages of anti-*Anisakis* positive (mean O.D. + 1 Standard Deviation) are shown below (Panel (**b**)), and the McNemar test was used (P = *p*-value). Correlations of anti-*Anisakis* antibodies between 1st (at admission) and 2nd (after 7–10 days) analyses are shown in Panel (**c**). The Spearman test was used. IgS: total immunoglobulins (Igs).

In the 2nd analysis (7–10 days after sepsis evolution), an increase in all isotypes of immunoglobulins against *Anisakis* was observed in septic shock patients, except IgE (Figure 1a). A significant increase in the positivity of anti-*Anisakis* IgG was observed in patients with septic shock. Furthermore, the percentage of IgM- and IgA-positive septic patients was close to significance (*p* < 0.07 and *p* = 0.05, respectively) (Figure 1b). A direct correlation was observed between first (at admission) and second (after 7–10 days) analyses in all anti-*Anisakis* isotypes of immunoglobulins (Figure 1c). Furthermore, we compared the antibody levels of patients with sepsis with those of the control group of healthy subjects (Figure S1). Anti-*Anisakis* IgG and IgA values were lower in healthy subjects than in patients with sepsis. However, IgM values were higher in the healthy controls.

### 3.2. Immunoglobulins Anti-Anisakis and Numbers of αβ and γδ T Cell Subsets and Mortality

**Figure 2:**
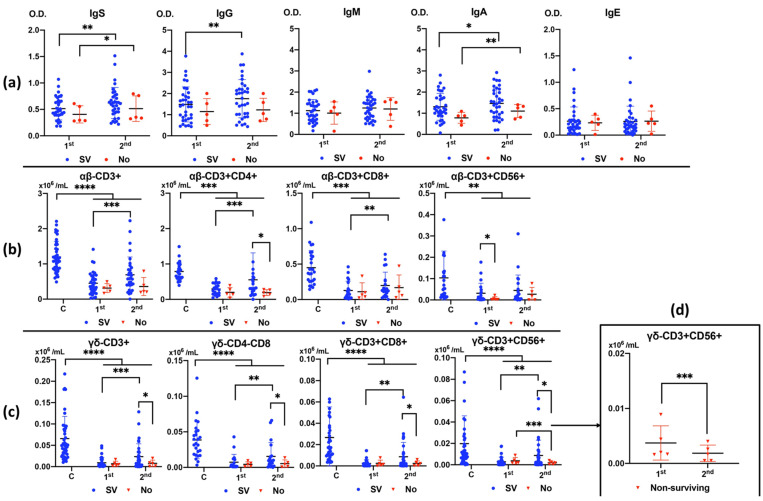
Differences in anti-*Anisakis* antibodies between 1st (at admission) and 2nd (after 7–10 days) analysis according to surviving (SV) and non-surviving (No) patients (Panel (**a**)). Values are expressed as means of Optical Density (O.D.). T bars denote standard deviation. Numbers of αβ and γδ T cell subsets (Panels (**b**,**c**)) are shown according to surviving (SV) and non-surviving (No) patients compared to control healthy subjects (Cs) between 1st (at admission) and 2nd (after 7–10 days) analyses. Differences in the number of CD3+CD56+ γδ T cells between 1st (at admission) and 2nd (after 7–10 days) analyses in non-surviving patients (Panel (**d**)). Values are expressed as means (×10^6^/mL) and T bars denote standard deviation. For the differences between 1st (at admission) and 2nd (after 7–10 days) analysis, the Wilcoxon test was used. For the differences between surviving and non-surviving patients, the Mann–Whitney U test was used. (**** *p* < 0.0001, *** *p* < 0.001, ** *p* < 0.01, and * *p* < 0.05). IgS: total immunoglobulins (Ig’s).

The mortality rate was 12% (N = 5). There was an increase in IgG and IgA anti-*Anisakis* antibodies during admission, first (at admission) to second (after 7–10 days), in surviving patients and an increase in IgA anti-*Anisakis* after 7–10 days of admission in non-surviving patients (Figure 2a).

All αβ and γδ T cell subsets (Figure 2b and Figure 2c, respectively) were greatly decreased in patients with sepsis in the first analysis (at admission), especially in the case of γδ T cells, compared to healthy controls. The number of T cells recovered in the second analysis (after 7–10 days) in surviving patients, whereas they did not recover in non-surviving patients, even when there was a decrease in CD3+CD56+ γδ T cell numbers (Figure 2d).

### 3.3. Apoptosis of αβ and γδ T Cell Subsets and Mortality

**Figure 3:**
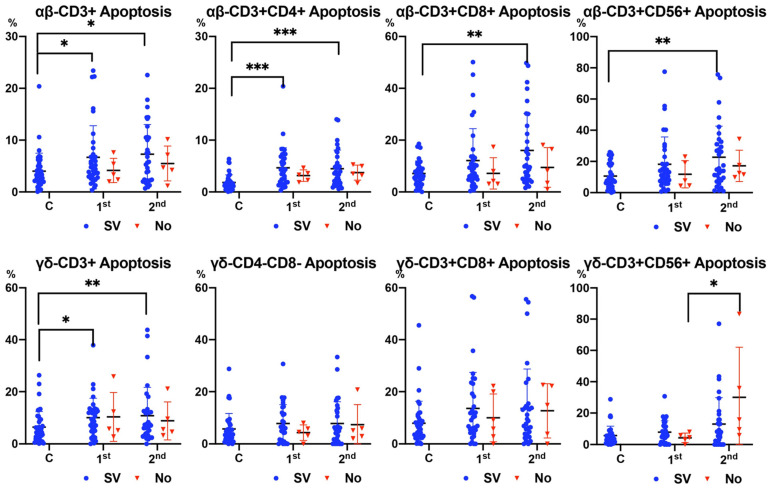
Apoptosis of αβ and γδ T cell subsets according to surviving (SV) and non-surviving (No) patients compared to control healthy subjects (Cs) between 1st (at admission) and 2nd (after 7–10 days) analysis. Values are expressed as percentages. For the differences between 1st (at admission) and 2nd (after 7–10 days) analyses, the Wilcoxon test was used. For the differences between surviving and non-surviving patients, the Mann–Whitney U test was used. (*** *p* < 0.001, ** *p* < 0.01, and * *p* < 0.05).

In surviving patients, Figure 3 shows increased apoptosis of all αβ T cell subsets and CD3+ γδ T cells in patients with sepsis compared to healthy subjects. There was a significant increase in apoptosis in the second analysis (after 7–10 days) compared with the first analysis (at admission) in the CD3+CD56+ γδ T cell subset only in non-surviving patients (Figure 3).

### 3.4. Differences in Anti-Anisakis Immunoglobulins According to Deficit of αβ and γδ T Cell Subsets in Surviving Septic Patients

**Figure 4:**
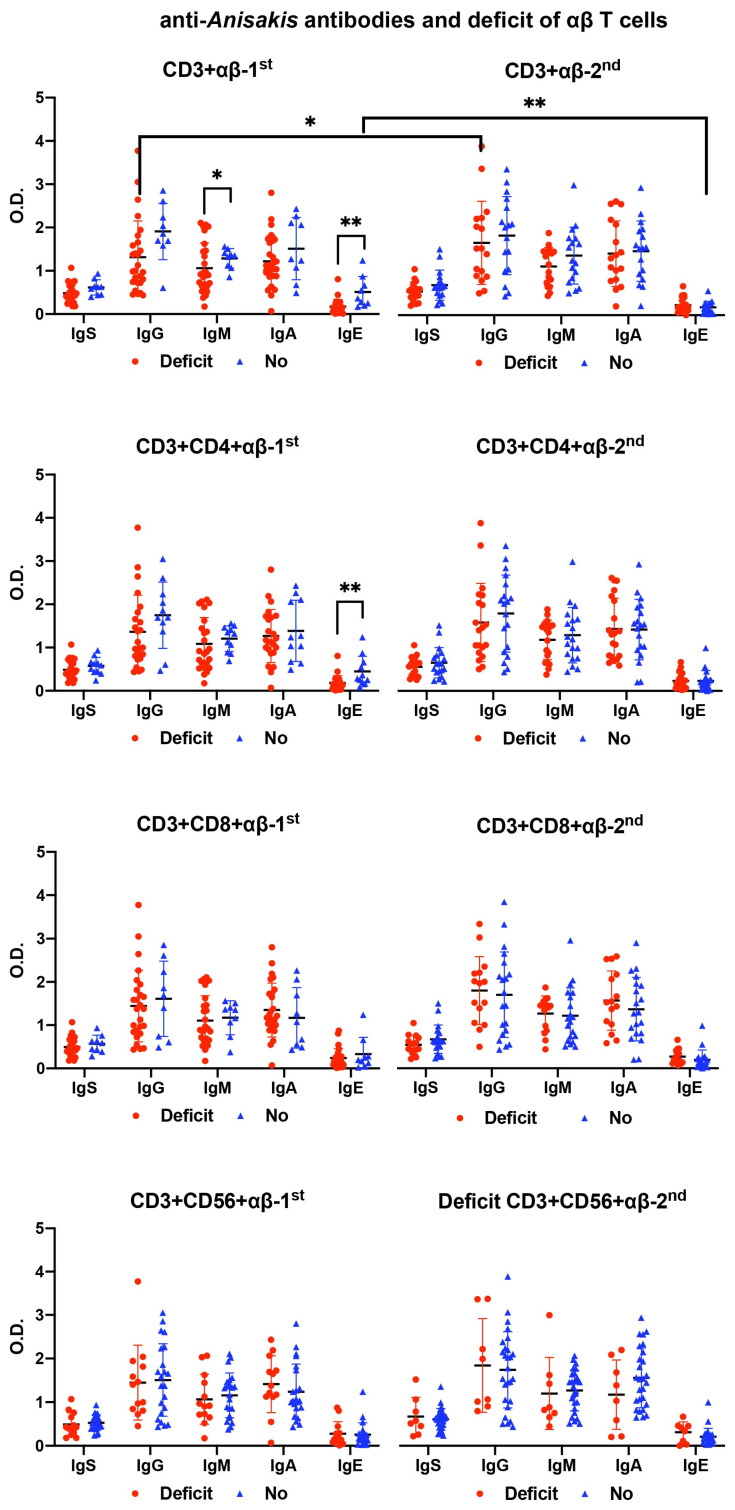
Differences in anti-*Anisakis* antibodies according to deficiency (Deficit) or no deficiency (No) of αβ T cell subsets in surviving septic patients (Mann–Whitney U test). For the differences between 1st (at admission) and 2nd (after 7–10 days) analyses, the Wilcoxon test was used. (** *p* < 0.01, and * *p* < 0.05). IgS: total immunoglobulins (Ig’s).

There was a decrease in IgM and IgE levels in septic patients with a deficiency of αβ T cell subsets at admission, specifically, a decrease in IgE levels in the case of CD4+ αβ deficiency. There was an increase in IgG and IgE levels in the second analysis of septic patients with of CD3+ αβ T cell deficiency at admission (Figure 4).

**Figure 5:**
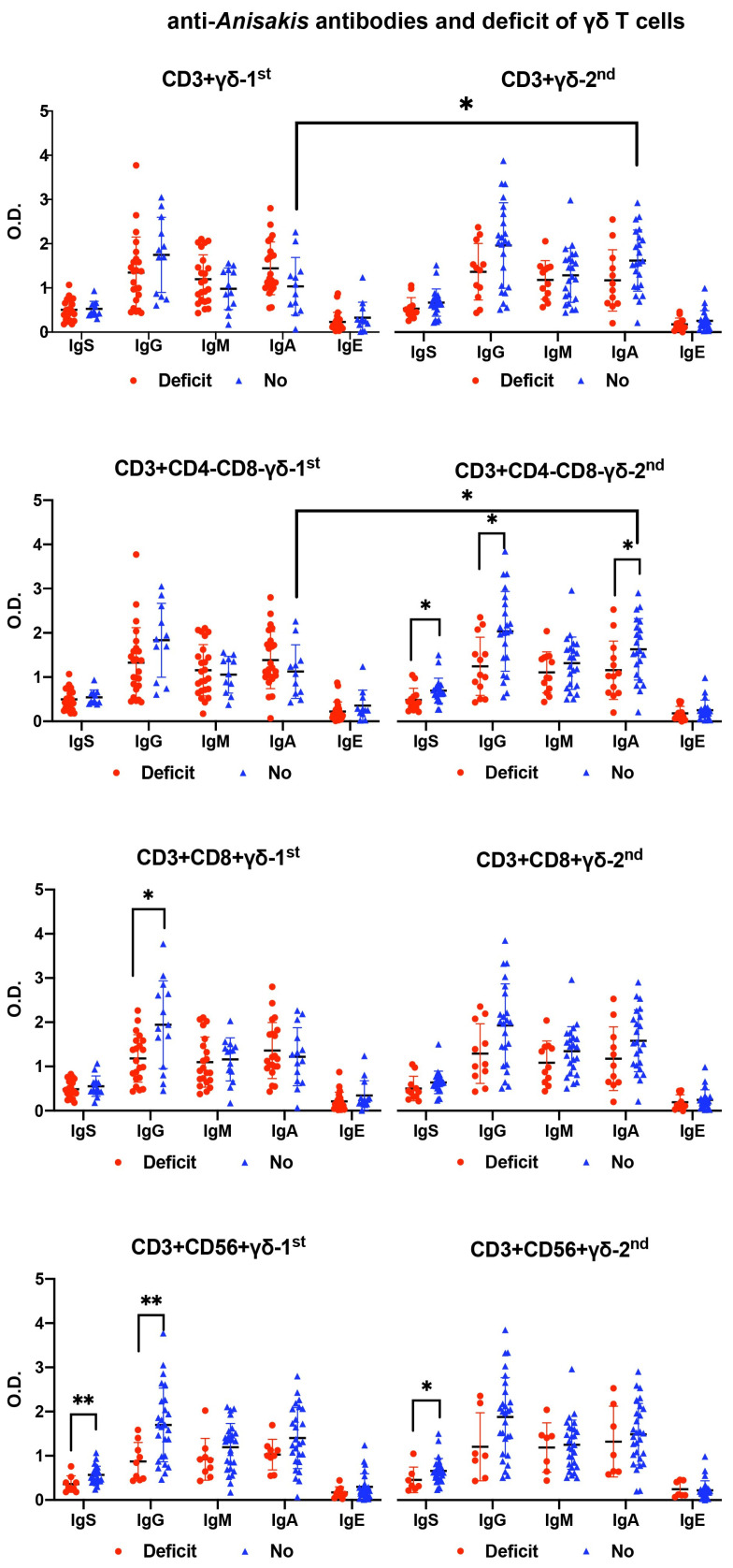
Differences in anti-*Anisakis* antibodies according to deficiency (Deficit) or no deficiency (No) of γδ T cell subsets in surviving septic patients (Mann–Whitney U test). For the differences between 1st (at admission) and 2nd (after 7–10 days) analysis, the Wilcoxon test was used. (** *p* < 0.01, and * *p* < 0.05). IgS: total immunoglobulins (Ig’s).

Patients with a deficiency in CD8+ and CD56+ γδ T cells at admission presented significantly lower levels of anti-*Anisakis* IgG. Patients without γδ T cell deficiency had increased their anti-*Anisakis* IgA levels 7–10 days after admission, which did not occur in patients with γδ T cell deficiency (Figure 5).

### 3.5. Correlations Between αβ-γδ T Cell Numbers with Anisakis Antibody Levels

Figure S2 shows significant correlations between anti-*Anisakis* antibody levels and numbers of αβ and γδ T cells in septic surviving or non-surviving patients between the first (at admission) and second (after 7–10 days) analyses. Surviving patients showed a direct relationship between IgG and IgE anti-*Anisakis* and γδ T cell levels at admission. In addition, the number of CD3+CD8+ αβ cells was positively related to anti-*Anisakis* IgE levels (Panel b). An inverse relationship was observed between anti-*Anisakis* IgG and the number of CD3+CD8+ γδ T cells in non-surviving patients. However, a direct relationship was observed between anti-*Anisakis* IgA and the number of CD3+CD8+ γδ T cells in non-surviving patients. There was also a negative relationship between the number of CD3+CD4+ αβ T cells and anti-*Anisakis* IgE in non-surviving patients (Panel b). A total of 7–10 days after admission (second analysis), in patients who did not survive, we observed the same relationship between anti-*Anisakis* antibody levels and the number of T cells. The relationship was positive between anti-*Anisakis* IgA levels and the number of CD3+CD4-CD8- γδ T cells and between anti-*Anisakis* IgE levels and the number of CD3+CD8+ αβ T cells. However, the relationship between anti-*Anisakis* IgM levels and the number of CD3+CD4-CD8- γδ T cells was negative (Panel c).

### 3.6. Correlations Between Apoptosis of αβ-γδ T Cells with Anti-Anisakis Antibody Levels

Figure S3 shows differences between the first analysis (at admission) and second analysis (after 7–10 days) according to the correlation between apoptosis of αβ-γδ T cells and anti-*Anisakis* antibody levels in surviving and non-surviving patients. In the first analysis (at admission), there was a negative relationship between the total antibody levels and the number of double-negative γδ T cells. Likewise, a negative correlation was observed between CD3+CD56+ γδ T cell apoptosis and anti-*Anisakis* IgM levels (Panel a). There was a negative relationship between apoptosis of CD3+CD4+ αβ T cells and anti-*Anisakis* IgA levels in non-surviving patients (Panel b). In the second analysis (after 7–10 days) of the surviving patients, there were positive correlations between IgA- and IgM-specific antibodies and apoptosis of double negative γδ T cells. Likewise, there was also a positive correlation between anti-*Anisakis* IgA and the apoptosis of CD3+CD56+ γδ T cells. In the second analysis (after 7–10 days) of the patients who did not survive, there was an inverse relationship between IgG anti-*Anisakis* levels and the apoptosis of both CD3+CD8+ αβ and γδ T cells. Likewise, there were also negative correlations between the apoptosis of CD3+CD56+ αβ T cells and both IgG- and IgA-specific antibody levels (Panel d).

## 4. Discussion

This is the first study that assesses the evolution of anti-*Anisakis* antibodies in the first days of the septic process and its relationship with αβ-γδ T cell immunity.

- Anti-*Anisakis* antibody evolution in septic patients:

As was evident, there was an increase in anti-*Anisakis* IgG, IgM, and IgA levels 7–10 days after hospital admission, and this increase was significant in patients with septic shock. Positivity against *Anisakis* was the highest in the case of specific IgG in septic shock 7–10 days after hospital admission, followed by anti-*Anisakis* IgA and IgM. Of note is the direct relationship of anti-*Anisakis* antibody levels between the first (at admission) and the second analysis (after 7–10 days). This indicates that the increase in specific antibody levels was progressive and uniform.

In a previous study, we demonstrated an increase in anti-*Anisakis* IgG levels in patients with septic shock compared to healthy control subjects. In that study, the levels of specific antibodies were analyzed only at admission [19]. In the present study, we verified that septic patients have higher levels of anti-*Anisakis* IgG, which suggests that they have had previous contact with the parasite. In other parasitic diseases, such as leishmaniosis, high anti-*Leishmania* IgG titers were correlated to mucosal leishmaniosis severity [23]. In our study, the greatest increase in specific IgG and IgA levels was observed in the most severely ill patients (septic shock). Likewise, the relationship between some nematodes and the enhancement of infectious and septic processes has been suggested, especially invasive bacterial infections of enteric origin [24,25,26,27,28,29]. This finding suggests that patients with parasitic infections are more susceptible to bacterial infections. This could be because of an immune deficiency that predisposes them to infection by parasites and bacteria. Some authors have indicated that granulomas induced by nematode larvae can create sites of nesting and bacterial proliferation that could cause sepsis [29]. Based on these facts, we hypothesized that *Anisakis* infection and the subsequent production of granulomas are predisposing factors for the appearance of subsequent bacterial sepsis.

The levels of IgA, IgG, and IgE anti-*Anisakis* increase one month after infection and remain high at six months [17]. However, patients previously immunized against *Anisakis* larval antigens can reach higher levels of specific IgG after a second contact, sooner than after the first contact, which justifies our results. Thus, the fact that patients with sepsis present increased levels of specific IgG suggests that they would have already had previous contact with *Anisakis* at least a month before the septic process. In contrast, specific IgM levels did not increase during hospital admission, which does not suggest recent or active infection. However, it is feasible that a possible previous infection with *Anisakis* larvae would have led to the formation of granulomas that could have created an environment conducive to subsequent infections (bacterial, fungal, or parasitic). This suggests that *Anisakis* infection could be a risk factor for subsequent sepsis, and the release of antigens encapsulated in granulomas could explain the increase in specific antibodies during the septic process.

On the other hand, it would also be interesting to study whether *Anisakis* larvae could be involved in the septic process in patients with negative cultures and in whom the pathogen causing sepsis has not been identified.

- Relationship between T cell subtypes and anti-*Anisakis* antibodies:

In this study, we also compared the increase in anti-*Anisakis* antibody levels in patients who survived sepsis with those who did not survive. We observed that both specific IgG and IgA levels increased in the second analysis of the patients who survived. This suggests that an increase in the immune response of antibodies against *Anisakis* is associated with survival. In contrast, IgA, the most common immunoglobulin in the mucosa, was also increased in non-surviving patients. Furthermore, CD3+CD8+ γδ T cells were indirectly associated with specific IgA levels in non-surviving patients upon admission. This suggests a relationship between the increase in specific IgA levels and decrease in the number of CD3+CD8+ γδ T cells. It could be thought that the decrease in mucosal CD8+ γδ cells favors the release of somatic antigens from dead larvae retained in the granulomas and the subsequent production of specific antibodies.

As in our previous study, the number of T cells, especially γδ subsets, was lower in patients with sepsis than in healthy subjects [6]. Furthermore, subjects who survived sepsis showed an increase in the number of T cells 7–10 days after the beginning of the process, while those who did not survive did not show this increase. Specifically, patients who did not survive sepsis had decreased CD3+CD56+ γδ T cells 7–10 days after sepsis onset. This observation coincided with an increase in apoptosis in this T cell subset in these patients.

T cell deficiency was related to a decrease in anti-*Anisakis* antibody levels. In patients with αβ T cell deficiency, there was a decrease in the levels of anti-*Anisakis* IgM and IgE. In contrast, in patients with deficiency of CD8+ and CD56+ γδ T cells, a statistically significant decrease in specific IgG was observed.

In this study, the relationship between γδ T cells and anti-*Anisakis* IgG was demonstrated. First, because there was a direct correlation between anti-*Anisakis* IgG and all subpopulations of γδ T cells, a relationship was not observed with αβ T cells. Second, the deficiency of γδ T cell subsets was related to a decrease in anti-*Anisakis* IgG levels, especially in the case of cytotoxic γδ T cells (CD8+ and CD56+). Furthermore, there was a direct correlation between γδ T cells and anti-*Anisakis* IgG, except in non-surviving patients, in which it was negative (CD3+CD8+ γδ T cells).

Patients with a deficiency in the double-negative γδ T cell population showed a decrease in both anti-*Anisakis* IgG and IgA antibodies in the second analysis (after 7–10 days). Notably, the double-negative population is the most frequent among all γδ T cells. As mentioned in the results, in patients who did not survive, there was a direct relationship between double-negative γδ T cells and IgA anti-*Anisakis* levels after 7–10 days of the evolution of sepsis. Contrarily, a negative relationship between IgM anti-*Anisakis* and double-negative γδ T cells after 7–10 days of admission in non-surviving patients was observed. *Anisakis* larvae stimulate the formation of granulomas composed of granulocytes and giant cells. Subsequently, mature granulomas composed of eosinophils, fibroblasts, and collagen will be generated. Finally, the dead larvae will be enclosed inside the granulomas formed mainly by connective tissue, eosinophils, and multinucleated giant cells leading to infection [16,18]. We previously demonstrated that previous contact with *Anisakis* was related to a decrease in all γδ T cell subsets in healthy individuals [30]. This suggests that a decrease in γδ T cells prevents the maintenance of granuloma integrity by contributing to the release of somatic antigens. This was confirmed because there was no increase in the specific IgE in the second analysis (after 7–10 days). For the production of anti-*Anisakis* IgE, ingestion of live larvae that release excretion–secretion products is necessary. In patients with sepsis, there was only an increase in the levels of anti-*Anisakis* IgM, IgG, and IgA, which can be stimulated by somatic antigens. It is logical to think that hospitalized septic shock patients could not ingest fish parasitized by live *Anisakis* larvae. Human dendritic cells differentiated in the presence of live larvae or crude extract were able to stimulate autologous CD4+ T cells [31]. In our patients, the antigens released by degenerated granulomas could be captured by dendritic cells and taken to the surrounding lymph nodes to stimulate cells previously sensitized by the antigens released by live larvae, generating a typical secondary Th2 response with antibody production.

## 5. Conclusions

In conclusion, the present work demonstrated an increase in anti-*Anisakis* IgG, IgM, and IgA levels during hospital admission in patients with severe sepsis. There was a direct relationship between the increase in specific antibodies between first (at admission) and second (after 7–10 days) analyses. During the evolution of sepsis, there was an increase in anti-*Anisakis* IgG and IgA levels in surviving patients and only specific IgA levels in non-survivors. The increase in anti-*Anisakis* IgA during hospital admission was related to non-survival due to sepsis. T cell deficiency was related to a decrease in anti-*Anisakis* antibodies. Specifically, the deficiency of αβ T cells was related to a decrease in anti-*Anisakis* IgM and IgE levels. Finally, the deficiency of CD8+ and CD56+ γδ T cells was related to lower levels of specific IgG.

Our results support the hypothesis that a previous *Anisakis* infection could be involved as a predisposing and favoring factor in the subsequent septic process, and could be related to those patients who present negative cultures in which the pathogen causing the infection has not been identified.

## Figures and Tables

**Table 1:** Characteristics of patients with sepsis.

	No. (%) of Patients		No. (%) of Patients
		- *Organic Failure*	
- *Stages of Sepsis*		Acute Heart Failure	32 (80.0)
Sepsis	11 (27.5)	Cardiovascular	29 (72.5)
Septic Shock	29 (72.5)	Acute Renal Failure	26 (65.0)
- *Diagnosis*		Acute Respiratory Failure	19 (47.5)
Urinary Tract Infections	15 (37.5)	Metabolic	18 (45.0)
Pneumonia	7 (17.5)	Acute Hepatic Failure	13 (32.5)
Peritonitis	6 (15.0)	Hematologic	10 (25.0)
Primary bacteremia	3 (7.5)	Neurologic	9 (22.5)
Acute Cholangitis	1 (2.5)	- *Analytical and Score Parameters*	Mean ± S.D.
Acute Cholecystitis	2 (5.0)	APACHE II Score	19.6 ± 7.6
Meningitis	2 (5.0)	SOFA Score	6.8 ± 3.4
Cellulitis	1 (2.5)	C-Reactive Protein (mg/l)	198.6 ± 137.2
Acute pancreatitis	1 (2.5)	Procalcitonin (ng/mL)	44.8 ± 69.6
Endocarditis	1 (2.5)	Lactic Acid (mg/dl)	3.1 ± 2.7
Gastroenterocolitis	1 (2.5)	- *In-Hospital Death*	5 (12.5)

## Data Availability

The data that support the findings of this study are available from the corresponding author(s) upon reasonable request.

## Supplementary Materials

The following supporting information can be downloaded at: https://www.mdpi.com/article/10.3390/antib13040096/s1, Figure S1. Differences in anti-Anisakis immunoglobulins between 1st and 2nd analysis in all septic patients (n = 40), septic patients (n = 11), septic shock patients (n = 29), and the control group (healthy subjects). Values are expressed as means of Optical Density (O.D.). T bars denote standard deviation. The Wilcoxon test was used. (*** *p* < 0.001, ** *p* < 0.01, and * *p* < 0.05); Figure S2. Significative correlations between anti-*Anisakis* antibodies and number of αβ and γδ T cell subsets in septic surviving (n = 35) (Panel a) and non-surviving (n = 5) patients (Panel b and c). 1st (at admission) and 2nd (after 7–10 days) analysis. Pearson and Spearman tests were used in the 1st (at admission) and 2nd (after 7–10 days) analysis, respectively; Figure S3. Significative correlations between anti-*Anisakis* antibodies and apoptosis of αβ and γδ T cell subsets in septic surviving (n = 35) (Panel a and c) and non-surviving (n = 5) patients (Panel b and d) for 1st (at admission) and 2nd (after 7–10 days) analysis. Pearson and Spearman tests were used in the 1st (at admission) and 2nd (after 7–10 days) analysis, respectively.
