# Supplementary material for: Increased Levels of Anti-Anisakis Antibodies During Hospital Admission in Septic Patients
Source: Antibodies (Basel). 2024 Nov 27;13(4):96. doi: 10.3390/antib13040096 (PMC11672462; doi:10.3390/antib13040096)
Supplement: Supplementary file 1 [file antibodies-13-00096-s001.zip › antibodies-3314975-supplementary.pdf]

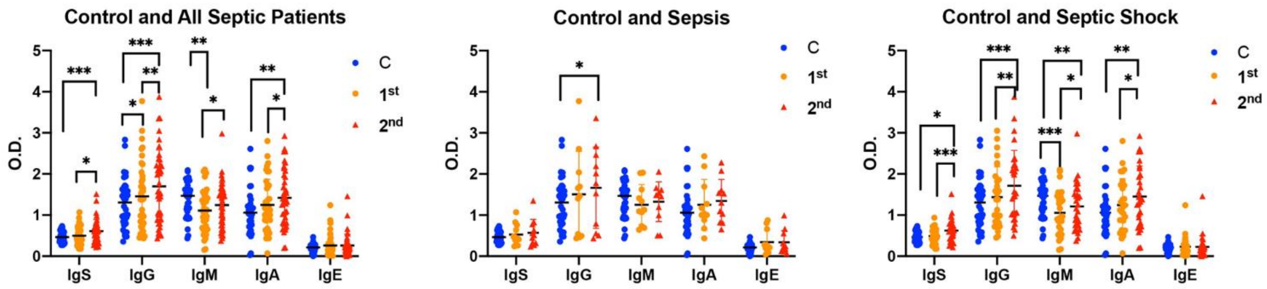

Figure S1. Differences in anti-*Anisakis* immunoglobulins between 1st and 2nd analysis in all septic patients (n=40), septic patients (n=11) septic shock patients (n=29) and the control group (healthy subjects). Values are expressed as means of Optical Density (O.D.). T bars denote standard deviation. Wilcoxon test was used. (***$p < 0.001$, **$p < 0.01$ and *$p < 0.05$). IgS: total immunoglobulins (Ig's).

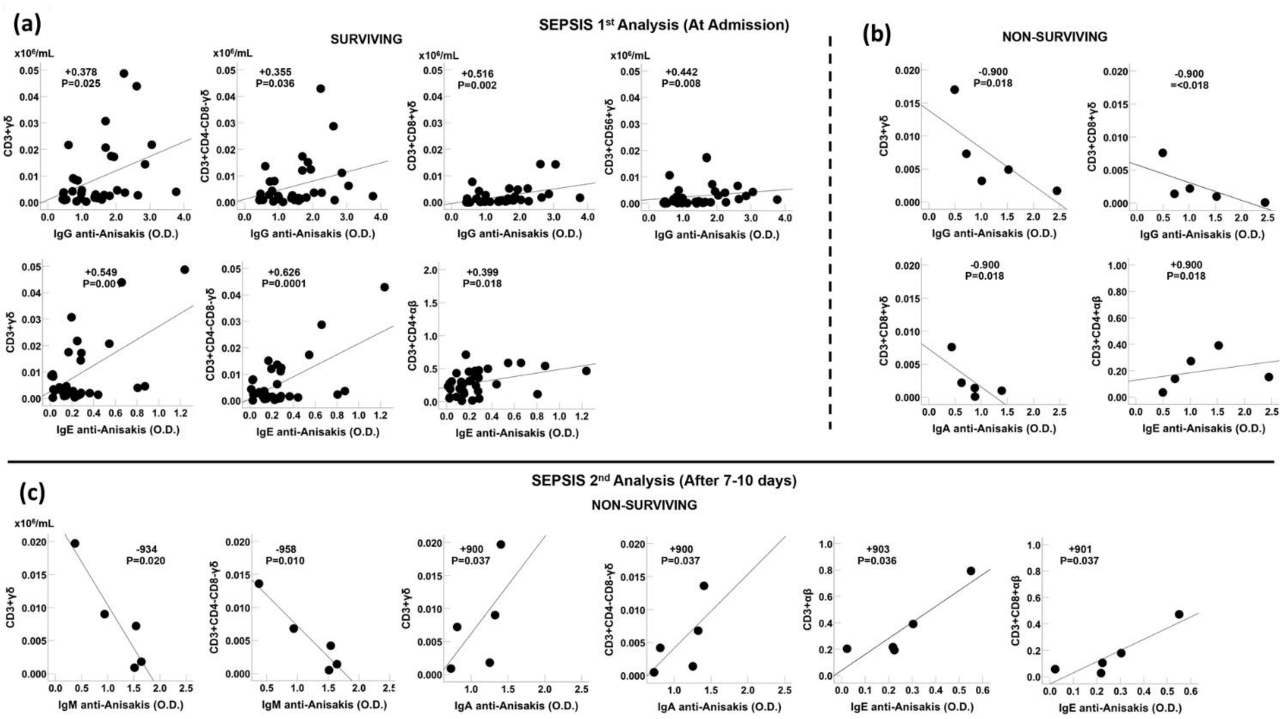

Figure S2. Significative correlations between anti-*Anisakis* antibodies and number of αβ and γδ T cell subsets in septic surviving (n=35) (Panel a) and non-surviving (n=5) patients (Panel b and c). 1st (at admission) and 2nd (after 7-10 days) analysis. Pearson and Spearman test were used in the 1st (at admission) and 2nd (after 7-10 days) analysis, respectively.

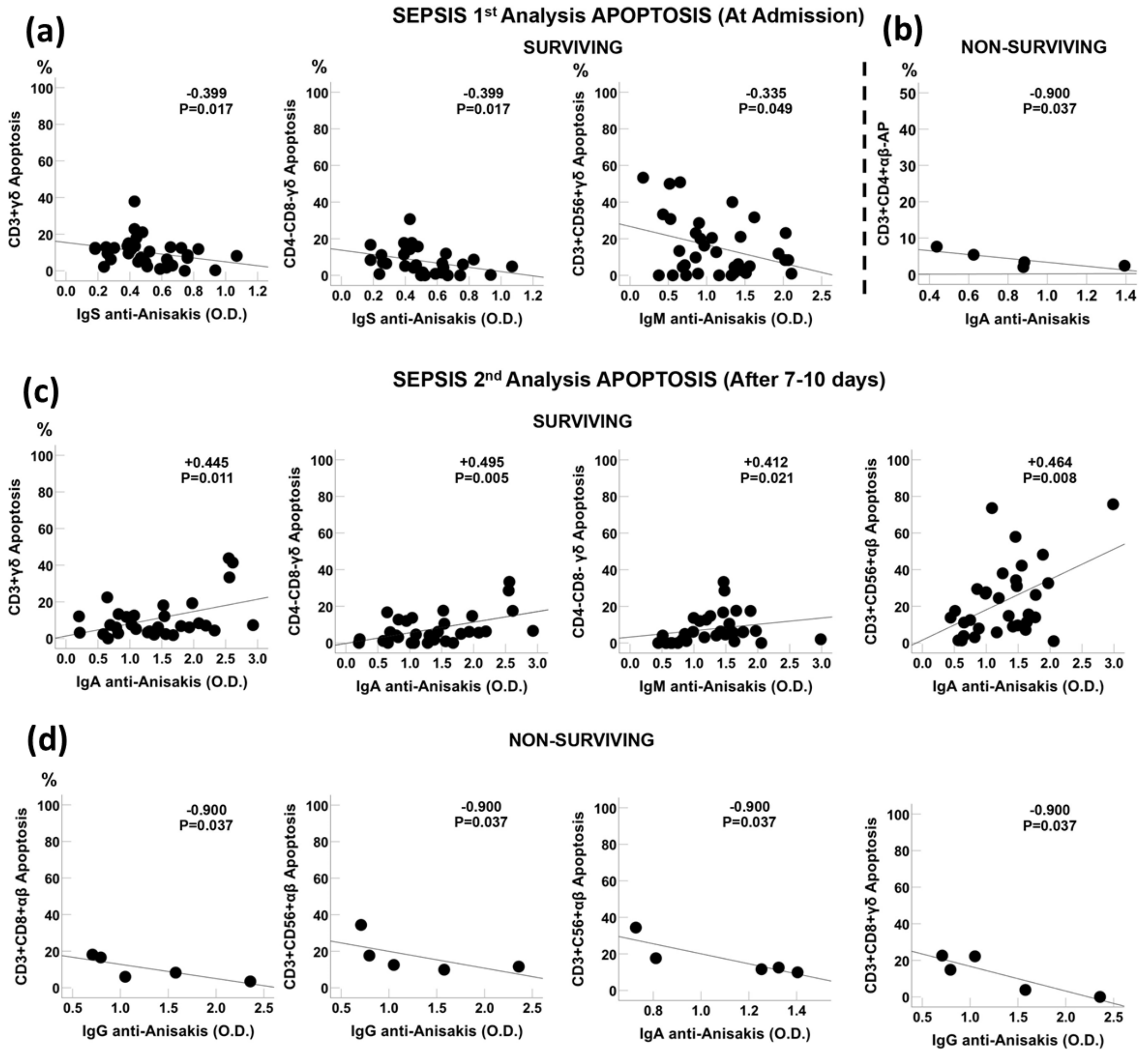

Figure S3. Significative correlations between anti-*Anisakis* antibodies and apoptosis of αβ and γδ T cell subsets in septic surviving (n=35) (Panel a and c) and non-surviving (n=5) patients (Panel b and d). 1st (at admission) and 2nd (after 7-10 days) analysis. Pearson and Spearman test were used in the 1st (at admission) and 2nd (after 7-10 days) analysis, respectively. IgS: total immunoglobulins (Ig's).
